# Heavy Metal Contents and Physical Parameters of *Aegiceras corniculatum, Brassica juncea*, and *Litchi chinensis* Honeys from Bangladesh

**DOI:** 10.1155/2015/720341

**Published:** 2015-11-04

**Authors:** Nandita Sarker, Muhammed Alamgir Zaman Chowdhury, Abu Naieum Muhammad Fakhruddin, Zeenath Fardous, Mohammed Moniruzzaman, Siew Hua Gan

**Affiliations:** ^1^Department of Environmental Sciences, Jahangirnagar University, Savar, Dhaka 1342, Bangladesh; ^2^Agrochemical and Environmental Research Division, Institute of Food and Radiation Biology, Bangladesh Atomic Energy Research Establishment, Savar, Dhaka 1349, Bangladesh; ^3^Department of Pharmacology, School of Medical Sciences, Universiti Sains Malaysia, 16150 Kubang Kerian, Kelantan, Malaysia; ^4^Human Genome Centre, School of Medical Sciences, Universiti Sains Malaysia, 16150 Kubang Kerian, Kelantan, Malaysia

## Abstract

The present study was undertaken to determine the heavy metal levels and the physicochemical parameters (pH, electrical conductivity (EC), and ash, moisture, and total sugar content) of honeys from Bangladesh. Three different floral honeys were investigated, namely, khalsi (*Aegiceras corniculatum*), mustard (*Brassica juncea*), and litchi (*Litchi chinensis*) honeys. The heavy metals in the honeys were determined by using a High Temperature Dry Oxidation method followed by Atomic Absorption Spectroscopy. The mean pH, EC, and ash, moisture, and total sugar contents of the investigated honeys were 3.6, 0.51 mS/cm, 0.18%, 18.83%, and 68.30%, respectively. Iron was the most abundant among all the investigated heavy metals, ranging from 13.51 to 15.44 mg/kg. The mean concentrations of Mn and Zn in the investigated honeys were 0.28 mg/kg and 2.99 mg/kg, respectively. Cd was below the detection limit, and lead was found in some honey samples, but their contents were below the recommended Maximum Acceptable Level. Cr was also found in all of the samples, but its concentration was within the limit. The physicochemical analysis of the honey samples yielded levels within the limits set by the international honey legislation, indicating that the honey samples were of good quality and had acceptable values for maturity, purity, and freshness.

## 1. Introduction

Honey is a natural product produced by honey bees, and the composition and properties of any individual honey sample depend highly on the type of flowers visited by the bees as well as on the climatic conditions in which the plants grow [1]. The mineral content of honey depends on the botanical origin and the geographical origin of the honey [2]. Honey is an indicator of environmental pollution [3]. The forage area of bees is very large (more than 7 km^2^ from the hive), and during the foraging process bees come into contact not only with air but also with soil and water [4]. The bees can come into contact with air and water that is contaminated with metals, which could be transported to the colony [5]. Moreover, air and water from industry and traffic contain heavy metals, which can also contaminate the bee colony and its products.

In Bangladesh, the possible emission of heavy metals occurs in various industrial sectors and activities, such as textiles and dyeing [6], ship-breaking areas, vehicular emissions, and agricultural runoff [7]. In Bangladesh, honey is produced and consumed on a large scale both commercially and noncommercially. Sundarbans, which is the largest mangrove forest in the world, is an ideal place for honey collectors. In recent years, the annual harvest has been established at approximately 200 metric tons of honey, with the honey produced from the Sundarbans area contributing to approximately 50% of the honey produced in Bangladesh. Large amounts of honey are also produced in different regions of the country. The plants and various agricultural crops grown in Bangladesh are excellent for bee foraging because bees are necessary for their pollination. Given their close proximity to urban development, the mangrove ecosystems are exposed to significant direct contaminant input [8].

To date, although honeys are produced in large quantities and are widely consumed in Bangladesh, very few data are available on heavy metal contamination in the honeys originating from different regions. Thus, the present work was aimed at determining the levels of seven metals, namely, cadmium (Cd), chromium (Cr), lead (Pb), copper (Cu), iron (Fe), zinc (Zn), and manganese (Mn), and six physicochemical parameters (pH, electrical conductivity (EC), ash content, moisture content, and sugar content) of various floral honeys that are produced in different areas of Bangladesh. The values were compared with two other honey samples (manuka honey from New Zealand and tualang honey from Malaysia). In addition, the honey was also investigated for correlation among individual constituents.

## 2. Materials and Methods

### 2.1. Chemicals and Reagents

Reference standard heavy metals such as Cd, Cr, Pb, Cu, Fe, Zn, and Mn were purchased from Kanto Chemical Co. Inc. (Tokyo, Japan). The chemicals used for digestion analysis were hydrochloric acid (HCl), nitric acid (HNO_3_), sulfuric acid (H_2_SO_4_), and hydrogen peroxide (H_2_O_2_); they were of analytical grade and they were purchased from Merck (*Darmstadt*, Germany).

### 2.2. Honey Samples

During this study, eighteen honey samples from three different floral types, namely, khalsi (*Aegiceras corniculatum*), mustard (*Brassica juncea*), and litchi (*Litchi chinensis*), were investigated (Table 1). The samples were collected directly from the comb and local apiary of different regions in Bangladesh between March 2013 and January 2014. At least 100 g of each honey sample was collected under sterile air in a tight glass bottle, labeled properly, and stored at 4-5°C until analysis. Two honey samples (manuka honey from New Zealand and tualang honey from Malaysia) were used as gold standards to compare their values with those from Bangladesh. The honeys were used as standards due to their well established chemical and biological properties. Manuka honey is a monofloral honey produced in New Zealand and Australia from the nectar of the manuka tree (*Leptospermum scoparium*) while tualang honey is a wild multifloral honey produced by the* Apis dorsata* bees. The bees collect nectar from plants and blossoms from a tall* Koompassia excelsa* tree, known locally as the “tualang tree,” in which the bees build their hives.

### 2.3. Physicochemical Parameters

#### 2.3.1. pH

The pH values of the honey samples were determined by using a pH meter (Cole-Parmer, Illinois, USA). A 10% (w/v) honey solution was prepared in fresh Milli-Q water. The pH of each honey was measured on the same day, and the experiments were conducted in duplicate for each sample.

#### 2.3.2. Ash Content

Approximately 1 g of honey sample was transferred into a porcelain crucible and heated for approximately 6 h at 450°C. Following complete ashing (the ash became white and grayish white), the samples were cooled in a desiccator and weighed [9]. The ash content was calculated by using the following formula:

(1) $$\begin{array}{c} Ash\left(\;\%\;\right)=\frac{\text{Weight of sample after ashing}}{\text{Weight of fresh sample}}\times 100. \end{array}$$

#### 2.3.3. Moisture Content

Approximately 3 g of honey was transferred into a porcelain crucible and heated for 3 h in an oven at 105°C. To ensure the complete removal of moisture, each crucible was reheated and weighed until the weight became constant. The moisture content was calculated by using the following formula:

(2) Moisture%=Weight of fresh sample−Weight of dry sampleWeight of fresh sample×100.

#### 2.3.4. Electrical Conductivity

The EC was determined according to the method established by the International Honey Commission [4]. The EC was measured at 20°C in a 20% (w/v) solution (dry matter basis) in distilled water by using a Hach conductivity meter. The result was expressed in millisiemens per centimeter (mS·cm^−1^).

#### 2.3.5. Total Sugar Content

The total sugar contents of the honeys were determined by using a refractometer (Delta Refractometer, Code 20–70, Bellingham + Stanley Ltd., England). The sugar content is represented by °Brix.

#### 2.3.6. Sample Preparation for Metal Analysis

The collected honey samples were prepared by a High Temperature Dry Oxidation method [9]. Each honey sample (1 g) was dried in a porcelain crucible at 100°C to its dry weight, which was then heated to 445°C for 6 h in an electrical furnace. After complete ashing, 3 mL of HNO_3_ was added, followed by acid evaporation on a hot plate at 100°C. Afterwards, 5 mL of HCl was added, and the volume was filled to 10 mL with distilled water (dilution 1 : 2). The solution was filtered and preserved in a refrigerator at 4-5°C until further metal analysis with an Atomic Absorption Spectrophotometer, model AA-6300, Shimadzu (Kyoto, Japan), equipped with a Shimadzu model GFA-EX7i graphite furnace atomizer to determine the heavy metals.

The measurement wavelengths for different heavy metals were as follows: Cd (228.67 nm), Cr (357.65 nm), Cu (324.57 nm), Mn (279.43 nm), Pb (217.35 nm), Zn (213.93 nm), and Fe (248.30 nm). Each sample was analyzed in triplicate. Two blanks were injected for each determination. For the calibration curve, standard solutions of each metal solution were prepared at different concentrations (0.0, 0.1, 1.0, 5.0, 10.0, 20.0, and 40.0 *μ*g/L) (Table 3). The metal analysis method used for the honeys was validated by using a recovery analysis. The percentage recoveries were calculated by using the following equation:

(3) $$\begin{array}{c} \text{Percentage recovery}=\left(\;\frac{CE}{CM}\times 100\;\right), \end{array}$$

where CE is the experimental concentration that was determined from the calibration curve and CM is the spiked concentration.

### 2.4. Statistical Analysis

The assays were performed in triplicate, and the results were expressed as the mean values with standard deviations (SD). Correlations were established by using Pearson's correlation coefficient (*r*) in bivariate linear correlations (*p* < 0.01). The correlations were calculated with SPSS version 18.0 (IBM Corporation, New York, USA), and the other analyses were performed with Microsoft Excel 2007.

## 3. Results and Discussion

### 3.1. Physicochemical Parameters

The mean pH value of the investigated honeys was 3.6, and there were large variations (Table 2). The honey samples collected from Sundarbans (khalsi) have relatively high pH values (3.69) in comparison with mustard (3.49) and litchi honey (3.62). The pH values of the investigated honeys were lower than that of manuka honey but similar to that of Malaysian tualang honey. The variations in the pH of the investigated honey occurred because of the variation in different acids and mineral contents in the honey [10]. In addition, floral differences may also contribute to the variability in the pH values. Our findings (mean pH: 3.60) were similar to the pH values reported for Malaysian acacia honey samples (mean pH: 3.43) [11]. However, our results were slightly lower than the ones reported previously for honey samples from Bangladesh [12].

Overall, the pH values of the investigated honey samples were within the acceptable range established by the Codex Alimentarius Commission [13], indicating the freshness of the honey samples and their potential use as good antibacterial agents [14]. The elevated acidity of honey reportedly occurs because of the fermentation of sugar, resulting in the conversion of sugar into organic acid; this acid is said to be responsible for honey's flavor and stability against microbial spoilage [15]. Thus, the low pH of honey creates unfavorable conditions for bacteria or any other microorganisms to grow.

The EC values of the investigated honeys from Bangladesh ranged from 0.32 to 0.74 mS/cm, which were within the allowable limit (lower than 0.8 mS/cm) set by the Codex Alimentarius and European legislation [16]. These values were also similar to values for previously reported honey samples from Bangladesh (0.2 to 0.8 mS/cm) [12] and India (0.33 to 0.68 mS/cm) [17] but lower than honeys from Portugal (0.63 to 0.65 mS/cm) [18]. In comparison, the EC values of the manuka and tualang honeys were 0.53 and 0.67 mS/cm, respectively. The differences in EC values shown here may be contributed by different amounts of minerals, organic acids, proteins, variability in floral origin, and the amount of plant pollen [19] for each type of honey.

The ash content provides an insight into the honey quality [20]. The investigated honey samples from Bangladesh had ash contents ranging from 0.07 to 0.24%, indicating that these honey samples had different micro and macro mineral contents, possibly because of their different botanical origins. All the investigated honey samples from Bangladesh had ash contents below 0.60%, which were lower than the contents of both manuka and tualang honeys. However, the mean ash levels were similar to levels in an earlier report on honeys (0.03 to 0.43%) from India [17] and Morocco (0.04 to 0.40%) [21] but lower than the levels in honeys (0.47 to 0.64%) from Portugal [18].

The moisture contents of honeys from Bangladesh varied between 17.89 and 19.58%. These values were within the maximum prescribed limit for honey moisture content (≤20%) in accordance with the Codex Alimentarius [13] and EU Council Directives [16]. Honey with a high water content is more likely to ferment, making its preservation and storage more difficult [22]. The moisture contents of the honeys in our study were similar to the moisture of previously reported honeys that were also from Bangladesh [12]. The moisture content range of pure honeys from Ireland was reportedly 16.10 to 23.36% according to Downey et al. [23], which is higher than our results. The low moisture content (<20%) in the honey samples from Bangladesh indicates that the investigated honey was ripe and suitable for storage.

**Figure 1:**
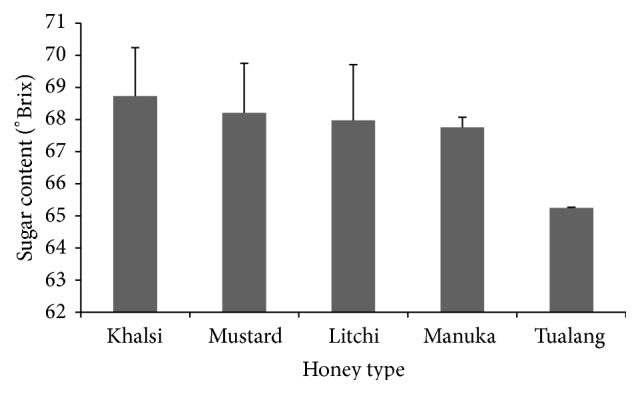
Sugar contents of the analyzed honey samples.

The total sugar contents of the honey samples from Bangladesh ranged from 65.51 to 71.18% (Figure 1). These values were similar to values for honeys from Estonia (62.88–78.32%) [24], but they were higher than values in honey samples from Bangladesh [12]. A relatively higher total sugar content (78.4–82.4%) was reported for honeys from India [17], which was higher than our results. According to the Codex Alimentarius [13], the total sugar contents of honey should be more than or equal to 60%, as they were in our findings, which clearly indicated the good quality of the investigated honey samples.

### 3.2. Metal Contents

Our study is the first extensive report on the metal contents of different types of honeys originating from Bangladesh (Table 4). The percentage recoveries of the investigated heavy metals in the honey samples were as follows: Cd (77.1%), Cr (93.4%), Cu (84.57%), Mn (76.51%), Pb (87.56%), Zn (77.42%), and Fe (87.56%). According to the European Commission [25], a method can be considered accurate and precise when the accuracy of the data is between 70 and 110%, and our result clearly supports the suitability and accuracy of our method for analyzing metals in honey.

Cd is of great concern, and Cd contamination usually has an anthropogenic source. Fredes and Montenegro [26] stated that higher Cd contents occur in honey that comes from beehives that are close to highways and processing equipment. According to the European legislation, the maximum limit for Cd content is 0.1 mg/kg [16]. With the exception of a single honey sample from Bangladesh, the Cd levels were generally below the detection limit for all honeys. The Cd contents for the manuka and tualang honeys were 0.06 mg/kg and 0.08 mg/kg, respectively.

Pb was detected in four mustard and three litchi honey samples, ranging from a level below the detection limit to 0.19 mg/kg. However, the levels were still within the recommended Maximum Acceptable Level (MAL) for Pb as suggested by the European Union at 1 mg/kg. The Pb concentration is usually related to environmental pollution [27]. The Pb concentration in the khalsi honey was below the detection limit, and similar results were also found for manuka and tualang honeys. The mean Cr concentrations in khalsi, mustard, and litchi were 0.76, 0.52, and 0.39 mg/kg, respectively.

The Cr concentrations in the honey samples from Bangladesh were lower than the concentrations in manuka (6.40 mg/kg) and tualang honeys (6.16 mg/kg). In addition, all the investigated honey samples in the present study had lower Cr contents than the honey samples from Switzerland as reported by Bogdanov et al. [28], at 0.003 to 0.329 g/kg. Higher Cr levels (8.1 ± 5.3 mg/kg) were also detected in honey samples from Urmia, Iran [29]. The lower Cr concentrations may indicate better quality honey in our samples. The variation in the Cr levels of different honeys from several countries may be related to geographical differences in the origins of the honeys [30]. Cr is usually directly deposited on the nectar via air or added by bees during pollination. Notably, the Cu concentration was higher in litchi honey (0.15 mg/kg) than in khalsi (0.11 mg/kg) and mustard honeys (0.09 mg/kg). However, the levels were lower than previously reported concentrations (0.12 to 0.34 mg/kg) in honey samples from Lithuania [31] and Hungary (0.02 to 0.78 mg/kg) [32]. By comparison, a relatively low Cu content was exhibited by both manuka and tualang honeys.

**Figure 2:**
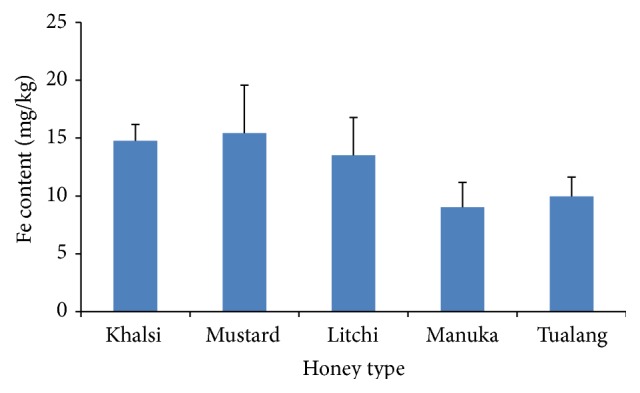
Fe contents of the analyzed honey samples.

Fe was the most abundant among all the metals analyzed during the present investigation. The mean Fe concentrations in mustard, khalsi, and litchi honeys were 15.44, 14.76, and 13.51 mg/kg, respectively, which were relatively similar and higher than the concentrations in manuka and tualang honey (Figure 2). Our result is similar to the level found in honeys from France at 11.03 mg/kg [33] but higher than that reported by Bogdanov et al. [28] at concentrations between 0.14 and 9.85 mg/kg and in honey samples from Hungary (0.11 to 2.86 mg/kg) [32]. The higher Fe concentration in the investigated honeys from Bangladesh may be related to the high iron availability in the botanical sources of mustard, litchi, and khalsi plants, which may be transferred to the honey.

**Figure 3:**
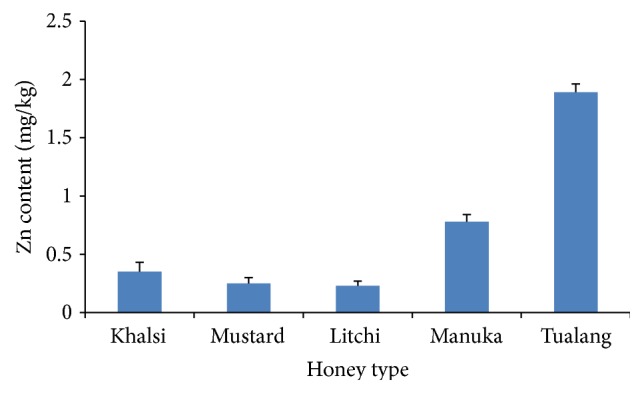
Zn contents of the analyzed honey samples.

The Zn concentrations of the investigated honeys from Bangladesh were between 0.23 and 0.35 mg/kg (Figure 3). However, a higher Zn concentration was exhibited by tualang honey (1.89 mg/kg), which had higher Zn than all the honey types. The Zn levels in the investigated honey samples were lower than the concentrations reported for Chilean honey, which ranged from 0.19 to 4.39 mg/kg [26]. The variation between the levels may be related to their different botanical and geographical sources. The amount of Zn in honey usually depends on the geographical location, botanical origin, and natural and anthropogenic sources [28].

Generally, Mn is present as a natural ingredient of minerals that are present in soils. The mean concentration of Mn was higher for khalsi honey (3.49 mg/kg) than mustard (2.78 mg/kg) and litchi (2.69 mg/kg) honeys, and it was 0.94 mg/kg for manuka and 1.02 mg/kg for tualang honeys. The Mn concentration was 0.03 mg/kg in honeys from Turkey [34] and 3.00 mg/kg for honeys from Nigeria [35].

Pearson correlation coefficients were determined between the metals within each type of honey originating from Bangladesh (Table 5). A strong positive correlation (*r* = 0.908) was observed between the Cr and Mn contents of the investigated honeys, indicating the relations between these two elements. However, Cr showed a negative correlation with Cu (*r* = −0.895). Moreover, statistically significant correlations were also observed between Pb and Cu (*r* = 0.997) and Pb and Mn (*r* = 0.997), indicating that the levels of these elements are interlinked with one another, which could be further investigated.

Hydroxymethylfurfural (HMF) is an important physical parameter of honey to determine its quality and freshness. However, HMF content of the currently investigated honeys was not determined due to unavailability of important equipment such as high performance liquid chromatography (HPLC) and gas chromatography mass spectrometry (GCMS) in the present investigation. Future studies are recommended including a higher number of honey samples originating from different parts of Bangladesh to measure the levels of different heavy metals and trace elements to analyze their levels and potential toxicity to the environment. Although the samples used in this investigation included mainly those from nonindustrialised areas, future study should also include analyzing the samples collected from industrialized areas for better comparison.

## 4. Conclusions

The levels of various metals in three different floral honeys from Bangladesh were reported for the first time in the present study. Several physical parameters were also determined, and our results indicated that all of the investigated honeys from Bangladesh are of good quality and the tested parameters met the requirements set by the International Honey Commission. The heavy metal concentrations were also within the limits, indicating their purity. Moreover, the higher concentrations of Fe in the investigated honeys signify that these honeys are a good source of these elements, which is very important for the human diet.

## Figures and Tables

**Table 1:** Floral type, source, and collection time of the investigated Bangladeshi honeys.

Honey type	Sample ID	Source location	Time of collection
Khalsi (*Aegiceras corniculatum*)	BH-1	East Satkhira range, Sundarbans	April 2013
	BH-2	East Satkhira range, Sundarbans	April 2013
	BH-3	East Satkhira range, Sundarbans	April 2013
	BH-4	East Satkhira range, Sundarbans	April 2013
	BH-5	East Satkhira range, Sundarbans	April 2013
	BH-6	East Satkhira range, Sundarbans	April 2013

Mustard (*Brassica juncea*)	BH-7	Birganj, Dinajpur	January 2014
	BH-8	Birganj, Dinajpur	January 2014
	BH-9	Singair, Manikganj	January 2014
	BH-10	Singair, Manikganj	January 2014
	BH-11	Madhupur, Tangail	January 2014
	BH-12	Madhupur, Tangail	January 2014

Litchi (*Litchi chinensis*)	BH-13	Dinajpur Sadar, Dinajpur	March 2013
	BH-14	Dinajpur Sadar, Dinajpur	March 2013
	BH-15	Manikganj Sadar, Manikganj	March 2013
	BH-16	Manikganj Sadar, Manikganj	March 2013
	BH-17	Kalaroa, Satkhira	April 2013
	BH-18	Kalaroa, Satkhira	April 2013

**Table 2:** Physicochemical parameters of the analyzed honeys in the present investigation.

Floral type	pH	Electrical conductivity (mS/cm)	Ash content (% w/w)	Moisture content (% w/w)
Khalsi (Sundarbans)	3.69 ± 0.04 (3.66–3.75)	0.74 ± 0.05 (0.67–0.80)	0.23 ± 0.01 (0.22–0.24)	18.55 ± 0.61 (18.09–19.45)
Mustard	3.49 ± 0.03 (3.46–3.54)	0.32 ± 0.03 (0.28–0.37)	0.10 ± 0.04 (0.07–0.16)	19.31 ± 0.58 (18.57–19.27)
Litchi	3.62 ± 0.09 (3.46–3.74)	0.47 ± 0.10 (0.32–0.55)	0.20 ± 0.03 (0.16–0.21)	18.64 ± 0.78 (17.89–19.58)
Mean ± SD	3.60 ± 0.10	0.51 ± 0.21	0.18 ± 0.07	18.83 ± 0.42
Manuka	3.94 ± 0.01	0.53 ± 0.03	0.27 ± 0.02	10.03 ± 0.15
Tualang	3.67 ± 0.02	0.67 ± 0.01	0.33 ± 0.01	14.81 ± 0.25

Mean ± SD (standard deviation) (min–max).

**Table 3:** AAS parameters.

Elements	Wavelength (nm)	Lamp intensity (mA)	Slit width (nm)	Linear range	Correlation coefficient (*r*)
Cd	228.67	8	0.7	80 *µ*g/L	0.998
Cr	357.65	10	0.7	80 *µ*g/L	0.997
Pb	217.35	10	0.7	80 *µ*g/L	0.998
Cu	324.57	6	0.7	40 *µ*g/L	0.999
Fe	248.30	12	0.2	6.0 mg/L	0.999
Zn	213.93	8	0.7	1.0 mg/L	0.994
Mn	279.43	10	0.2	80 *µ*g/L	0.998

**Table 4:** Metals contents in the analyzed honeys samples.

Floral type	Cd (mg/kg)	Cr (mg/kg)	Pb (mg/kg)	Cu (mg/kg)	Mn (mg/kg)
Khalsi (Sundarbans)	BDL	0.76 ± 0.18 (0.47–0.90)	BDL	0.11 ± 0.10 (0.01–0.23)	3.49 ± 0.98 (2.25–4.59)
Mustard	BDL	0.52 ± 0.29 (0.13–0.81)	0.10 ± 0.07 (BDL–0.19)	0.09 ± 0.04 (0.03–0.14)	2.78 ± 1.26 (1.46–5.10)
Litchi	0.01 ± 0.00 (BDL–0.01)	0.39 ± 0.32 (0.10–0.82)	0.16 ± 0.04 (BDL–0.19)	0.15 ± 0.06 (0.06–0.22)	2.69 ± 1.66 (1.13–5.19)
Range	BDL–0.01	0.39–0.52	BDL–0.16	0.09–0.15	2.69–3.49
Manuka	0.06	6.40	BDL	0.30	0.94
Tualang	0.08	6.16	BDL	0.41	1.02

BDL: below detection limit.

**Table 5:** Pearson correlation coefficients between metals in the investigated honeys.

	Cr	Cu	Zn	Fe	Mn
Cr	1.000	0.653	0.001	−0.268	0.908^*∗*^
Cu	0.653	1.000	0.524	−0.245	0.811
Zn	0.001	0.524	1.000	−0.422	0.099
Fe	−0.268	−0.245	−0.422	1.000	−0.390
Mn	0.908^*∗*^	0.811	0.099	−0.390	1.000

^*∗*^Correlation is significant at the 0.05 level (2-tailed).
